# Allergens Induce the Release of Lactoferrin by Neutrophils from Asthmatic Patients

**DOI:** 10.1371/journal.pone.0141278

**Published:** 2015-10-21

**Authors:** Lourdes Fernández-Delgado, Antonio Vega-Rioja, Inmaculada Ventura, Cristina Chamorro, Rocío Aroca, Manuel Prados, Pedro Bobadilla, David Rodríguez, Ricardo Palacios, Javier Monteseirín

**Affiliations:** 1 Unidad de Gestión Clínica de Alergia Intercentros, Hospital Universitario Virgen Macarena, Sevilla, Spain; 2 Departamento de Medicina. Facultad de Medicina. Universidad de Sevilla, Sevilla, Spain; 3 Servicio de Alergia, Hospital Infanta Elena, Badajoz, Spain; 4 Laboratorios Diater, Madrid, Spain; The Hospital for Sick Children and The University of Toronto, CANADA

## Abstract

**Background:**

Despite the evidence that Lactoferrin (Lf) is involved in allergic asthma processes, it is unknown whether neutrophils can be one of the main cellular sources of this key inflammatory mediator directly in response of an IgE mediated stimulus. The present study was undertaken to analyze this question.

**Methods:**

Neutrophils from healthy subjects (n = 34) and neutrophils from allergic asthmatic patients (n = 102) were challenged *in vitro* with specific allergens to which the patients were sensitized, PAF, or agonist mAbs against IgE-receptors, and the levels of Lf were measured in the culture supernatant. The levels of serum IgE together with the severity of symptoms were also analyzed.

**Results:**

Lf was released into the culture supernatant of neutrophils from allergic asthmatic patients in response to allergens and PAF. This response was highly allergen-specific, and did not happen in neutrophils from healthy donors. Allergen effect was mimicked by Abs against FcεRI and galectin-3 but not by FcεRII. The levels of released Lf correlated well with the levels of serum specific IgE and severity of asthma symptoms. These observations represent a novel view of neutrophils as an important source of Lf in allergic asthma. Importantly, the levels of released Lf by neutrophils could therefore be used to evaluate disease severity in allergic asthmatic patients.

## Introduction

Lactoferrin (Lf) is a protein involved in a large array of immune system activities in mammals that all lead to host protective effects [1]. In neutrophils, Lf is synthesized and stored in the secretory granules waiting for an external signal to be released; which is provided within the inflamed tissues. There, Lf is massively released, so that its iron-scavenging properties can be directed against microbes together with its direct microbicidal activity. The presence of high levels of Lf in inflammatory diseases indicates a possible use of Lf as a clinical marker [1]. Therefore, Lf clearly belongs to the innate nonspecific immune system, but also acts as a modulator of the inflammatory process. Lf binds to neutrophil membranes and promotes the activation and phagocytosis of neutrophils [2]. Lf was also reported as a promoter of motility, superoxide production, and release of proinflammatory molecules such as nitric oxide, Tumour Necrosis Factor-α (TNF-α), and Interleukin-8 (IL-8) from human neutrophils, monocytes and macrophages [1]. It has also been reported to act *in vitro* as a chemo-attractant for human neutrophils [3], and other cells [4]. Since inflammation may cause harmful systemic effects, there is a crucial need to regulate the immune process so that the response is commensurate. It is assumed that Lf, among others, exerts such a regulation of the immune response. The LPS-binding ability of Lf contributes to downregulating the activity and recruitment of innate immune cells. Lf was also shown to have anti-inflammatory properties, mainly by preventing the production and release of cytokines that induce recruitment and activation of immune cells at inflammatory sites [1]. The ability of Lf to bind iron makes the protein also a powerful anti-oxidant [5]. Thus, Lf may chelate ferric ion and prevent the formation of hydroxyl radicals and subsequent lipid peroxidation [6]. Our laboratory has shown that neutrophils from allergic patients release ROS in response to allergens in an IgE-mediated mechanism [7]. This mechanism was also involved in the induction of the expression of the key inflammatory enzyme cyclooxygenase-2, a process which requires formation of hydroxyl radicals through the Fenton reaction.

In regard to allergy, Lf also seems to play important anti-inflammatory roles. Allergy is a process that involves the activation of lymphocytes, macrophages, mast cells, basophils, eosinophils, neutrophils and others [7]. Interestingly, Lf is overexpressed in patients with allergies [8], and *in vivo* studies showed Lf protection against skin and lung allergies [8, 9]. Furthermore, the ability of Lf to destabilize tryptase, chymase, and cathepsin G, potent proinflammatory proteases released from mast cells, has been demonstrated [10–12]. These authors also showed *in vitro* an inhibition of anti-IgE induced histamine and tryptase release from human mast cells by Lf [8]. Finally, Lf decreases the recruitment of eosinophils [13], and reduces pollen antigen-induced allergic airway inflammation in a murine model of asthma [14]. There are 3 defined types of IgE receptors, all previously described in neutrophils (FcεRI, FcεRII/CD23, and galectin-3) [7]. We have previously shown that neutrophils isolated from allergic patients produce a functional response to those Ags that produce clinical symptoms [7]. There is increasing evidence of the participation of neutrophils in allergic processes in general, and in asthma in particular [7, 15, 16]. Despite the evidence that Lf is involved in asthma allergic processes, it is unknown whether neutrophils can be one of the main cellular sources of this key inflammatory mediator directly in response of an IgE-mediated stimulus. Here we show for the first time the ability of human neutrophils to release Lf in response to allergens which induce positive skin prick tests and specific IgE in asthmatic patients. The amount of released Lf was correlated with serum specific IgE levels and the severity of allergic asthma symptoms.

## Methods

### Ethics Statement

The Hospital Universitario Virgen Macarena ethics committee approved the study and each subject gave written informed consent **(Ref: C.I. 1772)**.

### Materials

The allergens (Ags) were commercially available Ag extracts, including D_1_ (*Dermatophagoides pteronyssinus*), G_3_ (*Dactylis glomerata*), T_9_ (*Olea europaea)*, and W_6_ (*Artemisia vulgaris*). They were purchased from Diater (Madrid, Spain). Platelet Activating Factor (PAF-C16, 1-o-hexadecyl-2-acetyl-sn-glycero-3-phosphorylcholine) was from Sigma (Madrid, Spain). Ficoll-Hypaque, phosphate-buffered saline (PBS), RPMI 1640, heat-inactivated foetal bovine serum, L-glutamine, penicillin and streptomycin were purchased from Lonza (Verviers, Belgium). Mouse monoclonal antibody (mAb) anti-FcεRI (α chain) clone AER-37 (CRA 1) was purchased from eBioscience (San Diego, CA, USA) [17]. Mouse mAb anti-FcεRII (CD23) clone 9P.25 was purchased from IZASA-Immunotech (Barcelona, Spain) [17]. Mouse mAb anti-galectin-3 clone A3A12 was from Abcam (Cambridge, UK) [17]. All culture reagents (including Ags) used in this work had endotoxin levels of ≤ 0.01 ng/ml, as verified by the Coatest *Limulus* lysate assay (Chromogenix, Mölndal, Sweden).

### Patients and controls

The studied groups included adult atopic patients with bronchial asthma, and healthy non-atopic volunteer controls. Asthma severity was classified based on a current guideline, into four groups: intermittent, mild persistent, moderate persistent, and severe persistent (S1 Table) [18]. The patients with allergic rhinitis were diagnosed on the basis of criteria previously described [19]. All subjects were lifelong non-smokers. Asthma was diagnosed on the basis of criteria previously described in detail [20]. The patients had positive skin prick test Diater and specific IgE (HYTEC 288, Hycor Biomedical Inc.-IZASA, Barcelona, Spain) to one common allergen (house-dust mites and pollens). The subjects received neither treatment nor specific hyposensitization. The asthmatic patients were not allowed to take short-acting β_2_-agonists within the 4 hours before challenge of neutrophils *in vitro*. Oral bronchodilators were withheld for 48 hours, and none of the subjects had taken corticosteroids, cromolyn sodium, or nedocromil sodium in the previous week. The healthy controls had no history of allergy or bronchial symptoms, and had negative skin prick test Diater and specific IgE (HYTEC 288) to a battery of inhalant allergens (house-dust mites, pollens, molds, and animal danders). No subjects had a history of infection within the previous 6 weeks.

### Cell isolation and culture

Highly purified human peripheral blood neutrophils were isolated as previously described [17]. Briefly, after isolation neutrophil preparations were further purified, depleting CD9^+^ cells (eosinophils), CD203c^+^ cells (basophils), and CD14^+^ cells (monocytes) using a magnetic cell-sorting system (MACS) by four rounds of incubation with mouse anti-human CD9, anti-human CD203c, and anti-human CD14 Abs, and then with anti-mouse IgG micromagnetic beads. The purity of the neutrophil preparations was determined as previously described [17]. This purification method reduced contaminating eosinophils to 0.001–0.004% of the final cell population. The purity of neutrophils was on average >99%. Monocytes, basophils and lymphocytes were not detected in the neutrophil preparations as previously showed [17]. Neutrophils were cultured in RPMI 1640 medium supplemented with 10% heat-inactivated foetal bovine serum, 2 mM L-glutamine, 100 U/ml penicillin, and 100 μg/ml streptomycin, and maintained at 37°C in an atmosphere of 95% O_2_ and 5% CO_2_. None of the reagents affected the viability of the cells at the concentrations used in this work, as confirmed by the Trypan Blue dye-exclusion test.

### Lung function

FEV_1_ was measured using a dry spirometer (Vitalograph, Buckingham, UK). The best value of three manoeuvres was expressed as a percentage of the predicted value.

### Dissociation of neutrophil-bound immunoglobulins

Ig molecules were dissociated from the cell-surface of neutrophils as described previously [21]. Briefly, after isolation, neutrophils were resuspended in 5 ml of a solution of 0.13 M NaCl, 0.005 M KCl, and 0.01 M lactic acid which was adjusted with 1N NaOH to pH 3.9 and incubated on ice for 5 min. An equal volume of PBS was then added to the treated cells, and the mixture was centrifuged, washed with PBS, and resuspended in RPMI 1640 medium. After treatment, neutrophils were cultured with the different agents.

### Analysis of released Lactoferrin

Lf was determined in the culture supernatant of 5 x 10^6^ cells cultured in 1 ml, using an ELISA kit from Oxis International Inc. (Portland, OR, USA), following manufacture's instructions. The sensitivity of the assay is 1 ng/ml. 5 μg/ml PAF was used as positive control [22].

### Statistical analysis

All statistical analyses were performed using GraphPad Prism version 4.00 for Windows (GraphPad Software, San Diego, CA, USA). Normality distribution was first examined using Shapiro-Wilk normality test before analysis of statistical significance. Data are expressed as means ± S.E.M. A one-way ANOVA was used to make comparisons between groups. Regression analysis was performed using Pearson rank correlation coefficients. A level of p< 0.05 was considered significant.

## Results

### Lf is released in response to allergens by human neutrophils from allergic asthmatic patients

**Fig 1:**
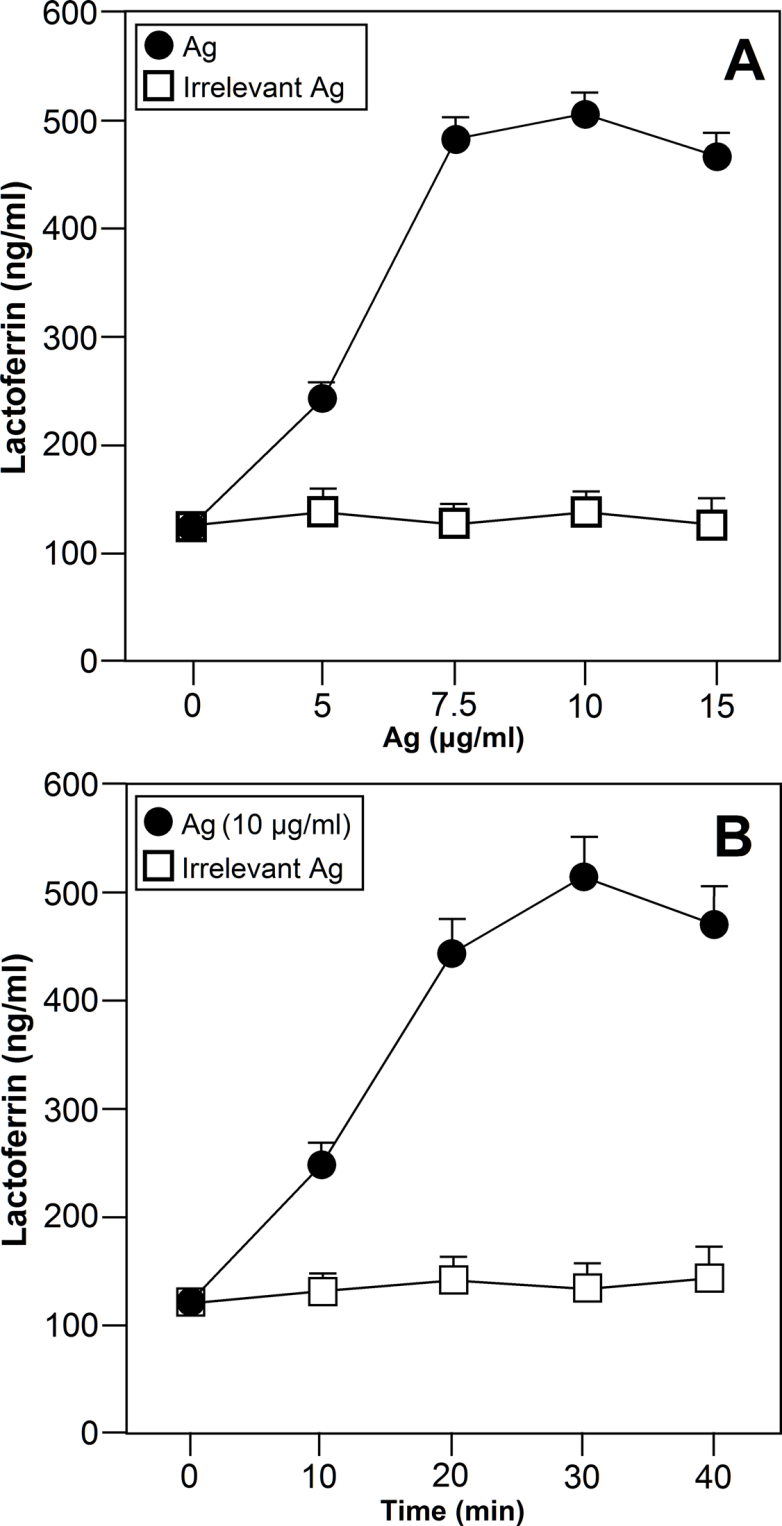
Ags induce the release of Lf by human neutrophils from allergic asthma patients. Neutrophils from allergic asthma patients were treated with an Ag to which the patient was sensitized or with an Ag to which the patient was not sensitized (irrelevant Ag, Ag to which the patients were not sensitized) for the indicated doses (n = 15)(A) and times (n = 15)(B). The level of Lf release was measured in the culture supernantant by ELISA. Data are expressed as mean± S.E.M.

**Fig 2:**
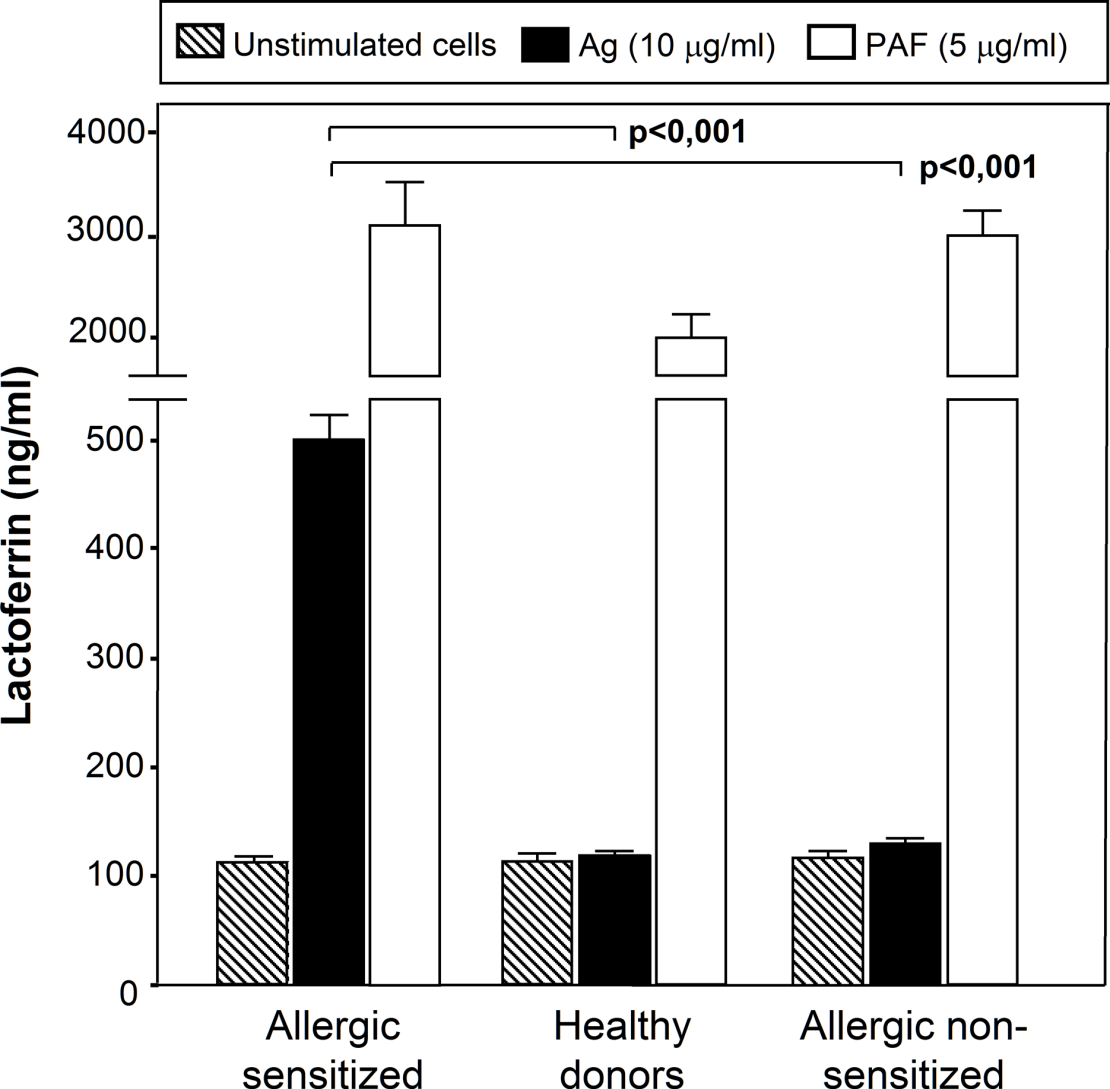
Specificity of the response to Ags. Neutrophils from allergic patients (Allergic sensitized) (n = 17) were left untreated or treated with PAF (5 μg/ml), or an Ag to which the patient was sensitized (10 μg/ml) for 30 min. Neutrophils from healthy donors (n = 17) were cultured with PAF (5 μg/ml) or an Ag (10 μg/ml) for 30 min. Neutrophils from allergic patients (Allergic non-sensitized) (n = 17) were left untreated or treated with PAF (5 μg/ml), or an Ag to which the patient was not sensitized (10 μg/ml) for 30 min. The level of Lf release was measured in the culture supernantant by ELISA. Data are expressed as mean ± S.E.M.

The effect of specific Ags to which the patients were sensitized upon Lf release into the culture supernatant was examined by ELISA. As shown in Fig 1A and 1B, the specific Ags to which the patients were sensitized (n = 15) stimulated Lf release by neutrophils as confirmed by ELISA. As also shown in Fig 1A and 1B, neutrophils released Lf in a dose- and time-dependent manner after exposure to the same Ags that induce clinical allergic symptoms in the patients. Lf release was detectable from 10 min and continued to increase up to 30 min. Lf release was detected at an Ag dose of 5 μg/ml, reaching the maximum at a dose of 10 μg/ml. No effect was observed at any dose of irrelevant Ags, Ags to which the patients were not sensitized. Fig 2 shows that neutrophils from allergic asthmatic patients (n = 17) cultured with an Ag to which the patients were sensitized released a significantly higher Lf amount than neutrophils from healthy donors (n = 17) or from asthmatic patients (n = 17) cultured with an Ag to which the patients were not sensitized (p<0.001). No statistical differences were observed between unstimulated cells, cells from healthy donors and cells from allergic subjects cultured in the presence of Ags to which the patients were not sensitized (Fig 2) (p = 0.818). Nevertheless, Lf release was clearly detected when neutrophils from donors from all groups were treated with PAF.

### Lf is released in response to Abs against FcεRI and galectin-3 by neutrophils from allergic asthmatic patients

**Fig 3:**
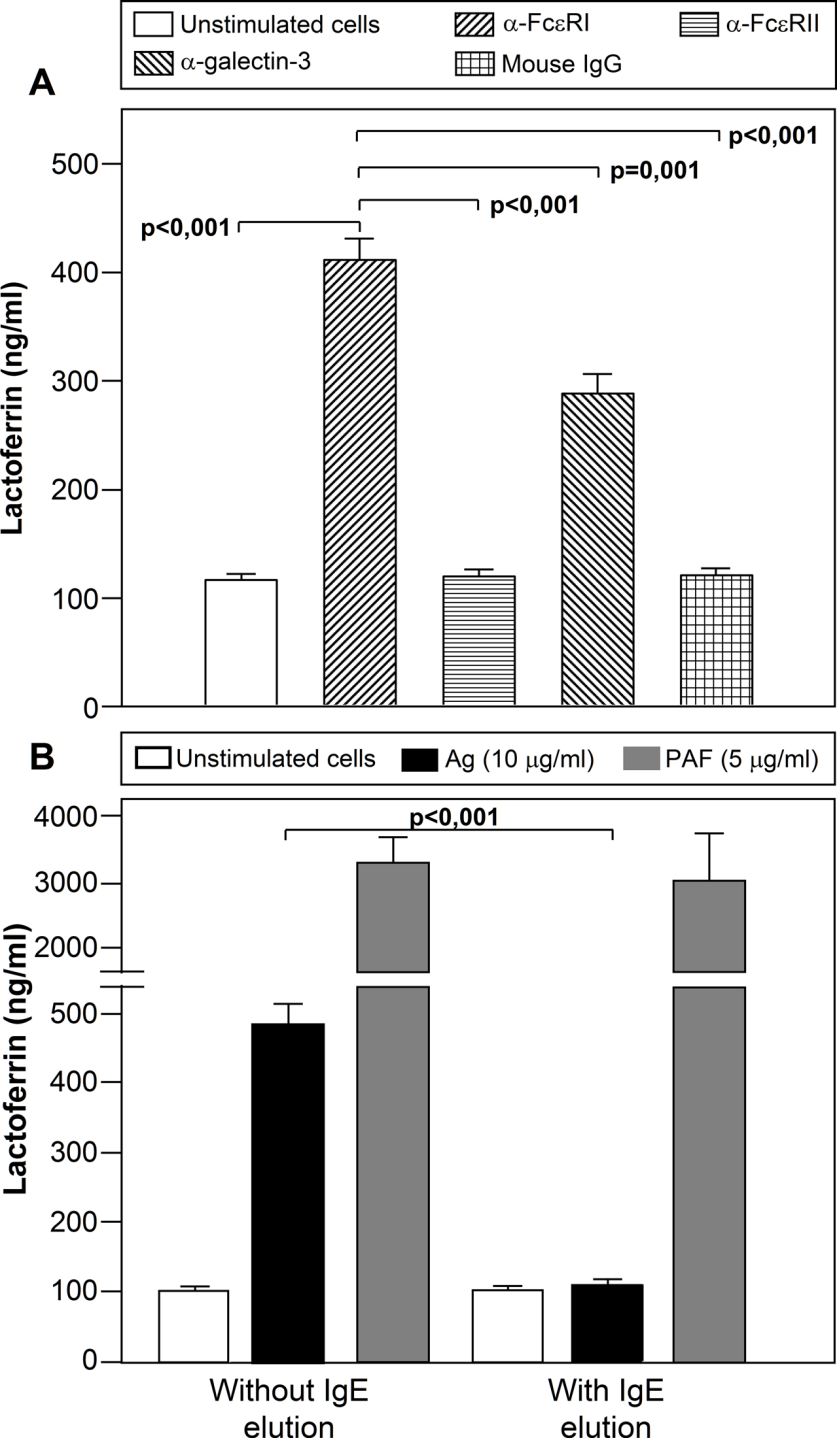
Involvement of IgE-receptors in the Lf release by neutrophils from allergic patients. (A) Neutrophils from allergic patients (n = 17) were left untreated or treated with anti-FcεRI (CRA1), anti-FcεRII (9P.25), anti-galectin-3 (A3A12) antibodies or mouse IgG_1_ at 5 μg/ml for 30 min, and Lf release was measured by ELISA in the culture supernatant. (B) Neutrophils from allergic patients (n = 17) were treated, where indicated, to elute the Igs from the cell surface, and then incubated with an Ag (10 μg/ml) to which the patient was sensitized or PAF (5μg/ml) for 30 min, and the level of Lf release was measured in the culture supernatant by ELISA. Data are expressed as mean± S.E.M.

Experiments were next performed to examine whether the engagement of IgE receptor/s could mimic the effect observed over the Lf release in response to Ags. The effect of agonist Abs against FcεRI (CRA1) or galectin-3 (A3A12) promoted the release of Lf into the culture medium (FcεRI>galectin-3 (p<0.001)), while anti-FcεRII (9P.25) had no effect (p = 0.788) (Fig 3). A non-specific mouse IgG Ab was used as a negative control.

We further investigated the participation of IgE in Ag-induced release. Lf release was not detected when IgE molecules were stripped from the neutrophil surface prior to Ag challenge, but was detected when neutrophils were incubated with PAF (Fig 3B).

**Fig 4:**
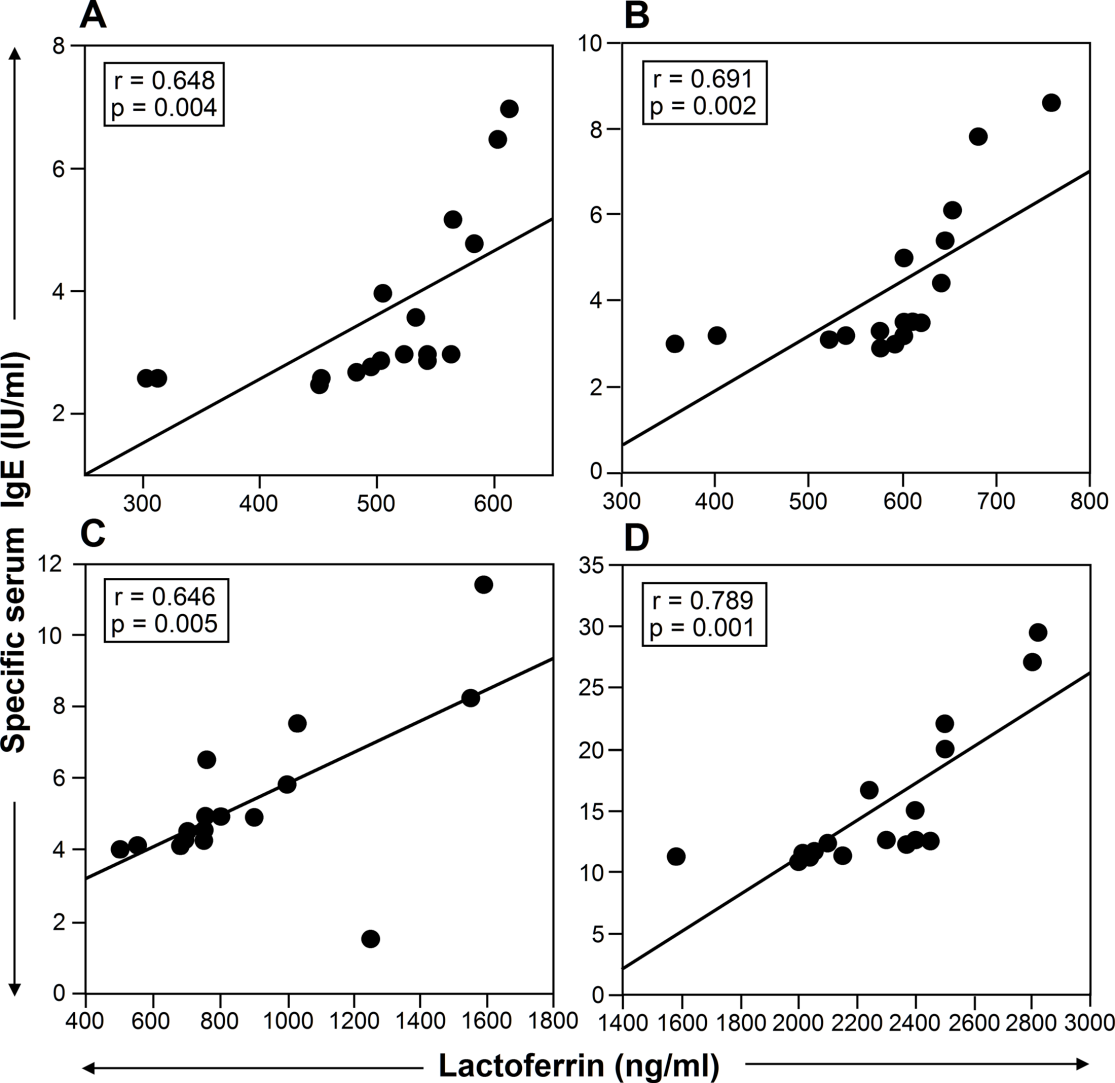
The amount of Lf release is correlated with the levels of serum specific IgE. The amount of Lf released by neutrophils from patients suffering from intermittent asthma without symptoms in the previous 4 weeks (n = 17) (A), mild persistent asthma (n = 17) (B), moderate asthma (n = 17) (C), and severe asthma (n = 17) (D) after incubation with allergens, was evaluated in relation to the patients’ levels of specific serum IgE. A significant correlation was observed between Lf release and specific serum IgE.

The amount of LF released by neutrophils from patients suffering of intermittent asthma without symptoms in the previous 4 weeks (n = 17) (Fig 4A), mild persistent asthma (n = 17) (Fig 4B), moderate asthma (n = 17) (Fig 4C), and severe asthma (n = 17) (Fig 4D), after incubation with allergens, was evaluated in relation to the patients’ levels of specific serum IgE. A significant correlation was observed between LF secretion and specific serum IgE (Fig 4).

**Fig 5:**
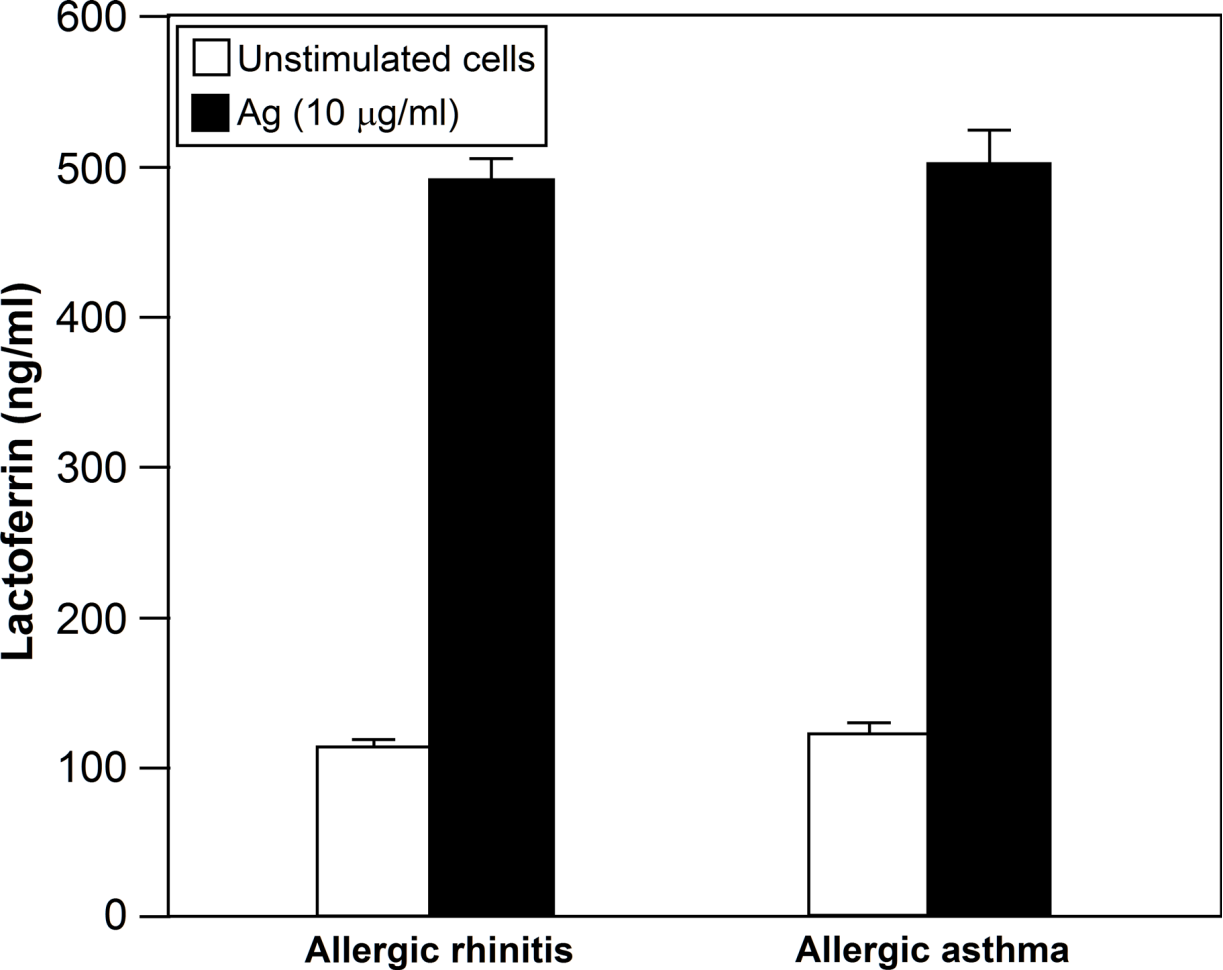
No statistical differences were observed between the levels of Lf released by neutrophils from patients suffering from intermittent asthma without symptoms in the 4 previous weeks (n = 17) and by neutrophils from patients suffering an extrinsic symptom-free rhinitis (n = 17) (p = 0.635).

Next experiments were performed to determine whether the release of Lf from neutrophils is related with asthma or rhinitis. No statistical differences were observed between the levels of released Lf by neutrophils from intermittent allergic asthma patients without symptoms in the 4 previous weeks (n = 17) and by neutrophils from allergic rhinitis patients without symptoms in the 4 previous weeks (n = 17) (p = 0.635) (Fig 5).

**Fig 6:**
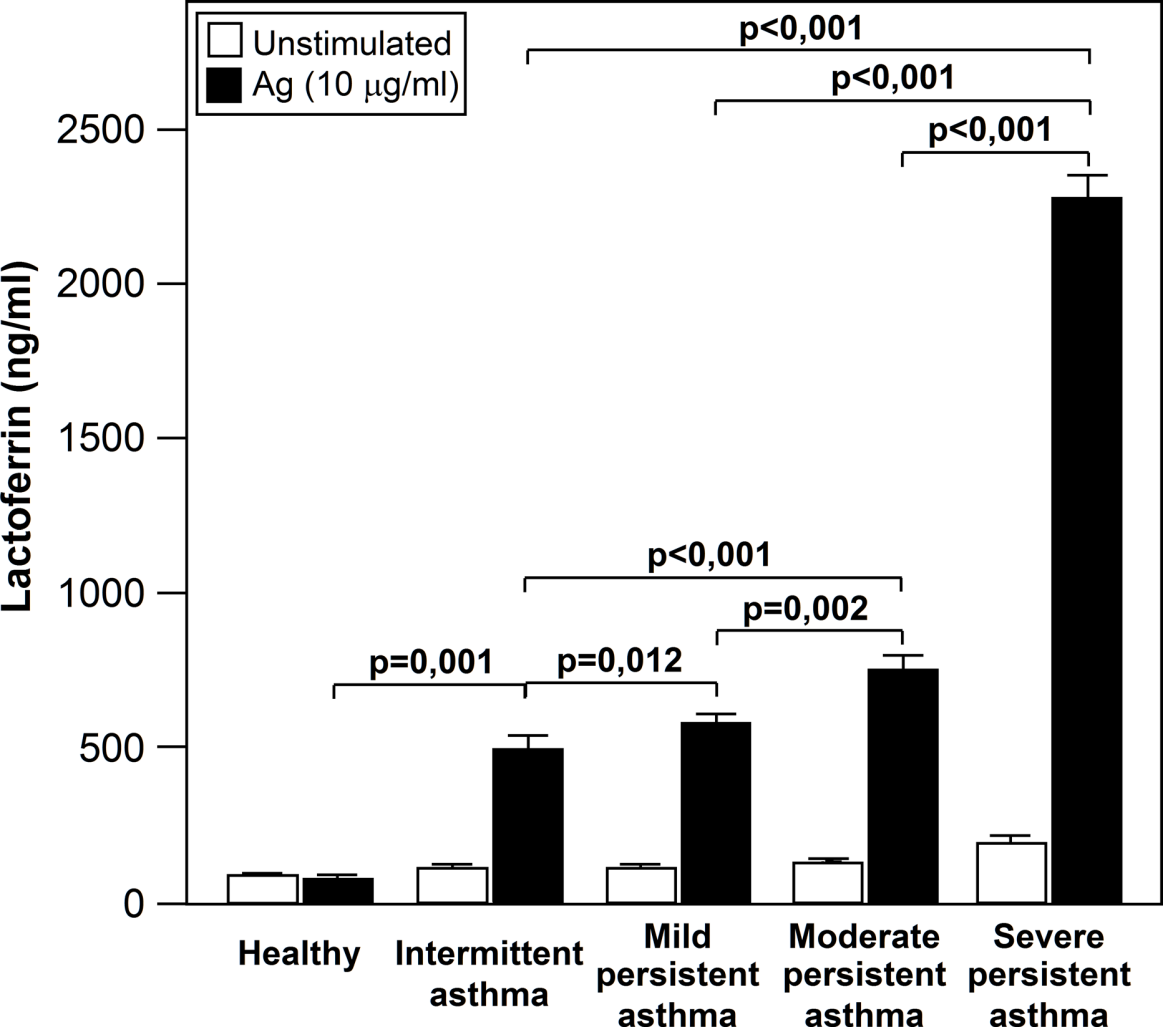
Lf release is increased with asthma severity. All groups of asthmatics had a higher Lf levels than those of control subjects. Significant between-group differences were detected for Lf, with a progressive increase in Lf concentration that was related to asthma severity.

We also analyzed whether Lf release was related with the level of symptoms (S1 Table). As shown in Fig 6, all groups of asthmatics had a higher Lf level than those of control subjects. Significant between-group differences were detected for Lf, with a progressive increase in Lf concentration that was related to asthma severity (Fig 6).

## Discussion

In this study, we have shown that in response to Ag challenge, neutrophils from allergic asthmatic patients release Lf in an Ig-dependent manner. This is supported by the following evidence: a) *in vitro* challenge of highly purified neutrophils from allergic asthmatic patients with Ags induced the release of Lf; b) anti-IgE receptors Abs mimic the effect of Ags upon Lf release, and c) Ag-mediated Lf release is cancelled by stripping the IgE molecules from the neutrophil surface, suggesting an involvement of IgE/IgE receptors.

Lf is secreted in an iron-free form from epithelial cells into most exocrine fluids, and particularly into milk [23]. To the best of our knowledge, none of the identified cellular sources of Lf were found to produce and release this important inflammatory mediator upon Ag challenge. Thus, the present data provide the first evidence that a human cell produces and releases Lf as a consequence of direct Ag challenge.

Lf is of particular relevance to allergic asthma and rhinitis [1, 24, 25]. Compared with normal subjects, increased concentrations of Lf have been detected in basal nasal washes of allergic rhinitis patients during pollen season [26] or Ag nasal challenge [27] and in induced sputum and bronchoalvelolar lavage fluid from asthmatic patients [28, 29]. Despite the evidence that Lactoferrin (Lf) is involved in asthma allergic processes, it is unknown whether neutrophils can be one of the main cellular sources of this key inflammatory mediator directly in response of an IgE-mediated stimulus. In the context of the allergic asthma, the involvement of neutrophils remains controversial. Asthma is an inflammatory disease with a complex immunopathology involving several different cell types and mediators. Eosinophils have been considered the most important cells in the pathophysiology of asthma and other allergic diseases. However, the interest in neutrophils as important mediators of the asthmatic airway inflammation has been renewed because they are the first cells to enter the airway in response to an allergen challenge and because the presence of airway eosinophilia does not fully explain this pathologic process (28). In this sense, the presence of the three IgE-receptors has been described in neutrophils [7]. Here we show that FcεRI and galectin-3 are involved in the process. We previously showed that these receptors are also involved in the release of IL-8, eosinophil cationic protein (ECP) and NFAT2 nuclear translocation [30–32]. The Ab against FcεRII/CD23 had no effect on Lf release, however we previously found that the same Ab has an effect on L-selectin [33] and CD66b expression [34], and in the production of histamine [17] and metalloproteinase-9 [35] by human neutrophils.

The role of galectin-3 is controversial. Recent studies in murine models using galectin-3 gene transfer indicate that galectin-3 is anti-inflammatory, however, a large number of *in vivo* and *in vitro* studies suggest that galectin-3 is pro-inflammatory [36]. Galectin-3-deficient mice develop significantly less airway hyperresponsiveness [37] and dermatitis [38] after allergen challenge and a lower T_H_2 response [37]. Galectin-3-deficient mast cells exhibit impaired mediator release and defective JNK expression [39]. However, lack of galectin-3 drives response to Paracoccidioides brasiliensis toward a T_H_2-biased immunity [40]. In this sense, we found that Lf is produced upon activation of galectin-3, and because Lf is involved in ROS production, and the release of other toxic mediators [41], our data suggest that it has a pro-inflammatory role.

In summary, we present evidence of a new mechanism of Lf release by human neutrophils from allergic asthmatic patients. In addition, the levels of serum specific IgE, and the severity of asthma symptoms correlated well with the Ag-mediated Lf release, therefore pointing out a possible use of Lf as a follow up tool to measure the progression of the allergic condition and/or the effectiveness of a treatment. In conclusion our data support a role of neutrophils as a source of Lf in Ag-induced rhinitis and asthmatic reactions.

## Supporting Information

S1 Table Demographic characteristics of the study groups. (PDF)
