# Supplementary material for: Allergens Induce the Release of Lactoferrin by Neutrophils from Asthmatic Patients
Source: PLoS One. 2015 Oct 21;10(10):e0141278. doi: 10.1371/journal.pone.0141278 (PMC4619071; doi:10.1371/journal.pone.0141278)
Supplement: S1 Table — (PDF) [file pone.0141278.s001.pdf]

S1 Table. Demographic characteristics of the study groups.

	Healthy	Intermittent Asthma	Mild Persistent Asthma	Moderate Persistent Asthma	Severe Persistent Asthma
n	17	17	17	17	17
Age (yrs.)	35 (18-36)	34 (19-34)	37 (20-45)	42 (38-48)	52 (47-62)
Sex (M/F)	9/8	7/10	9/8	5/12	6/11
Atopic	0	17	17	17	17
FEV_1 (% predicted)	100 (95-104)	101(98-104)	88 (80-96)	82 (79-90)	58 (50-64)
Lactoferrin (ng/ml)	119±6	501±21	584±23	762±46	2293±788
